# Ferroptosis in neurodegenerative diseases: potential mechanisms of exercise intervention

**DOI:** 10.3389/fcell.2025.1622544

**Published:** 2025-06-30

**Authors:** Shaokai Tang, Jianhua Zhang, Jiawei Chen, Zeng Zhou, Qinqin Lin

**Affiliations:** ^1^ College of Sports Science, Jishou University, Jishou, China; ^2^ School of Physical Education and Art, Hunan University of Medicine, Huaihua, China; ^3^ Hunan Mechanical and Electrical Polytechnic, Student Affairs Office, Changsha, China; ^4^ Department of Physical Education, Central South University, Changsha, China; ^5^ School of Physical Education, Yanshan University, Qinhuangdao, China

**Keywords:** exercise, ferroptosis, neurodegenerative disease, exerkine, iron metabolism

## Abstract

Neurodegenerative diseases represent a major global cause of mortality and disability. These disorders are characterized by complex pathogenesis and currently lack effective therapeutic strategies. Iron, a vital trace element for normal brain function, has been implicated in the pathogenesis of neurodegenerative diseases via the ferroptosis pathway. Emerging evidence indicates that exercise can suppress ferroptosis directly or indirectly by regulating iron metabolism, oxidative stress, and exerkine expression, thereby conferring neuroprotection. This review summarizes current insights into the role of ferroptosis in neurodegenerative diseases and explores the mechanisms by which exercise modulates the ferroptosis pathway, offering a scientific rationale for exercise-based interventions in brain health.

## 1 Introduction

Neurodegenerative diseases (NDDs) comprise a heterogeneous group of disorders characterized by the progressive loss of neurons or myelin in the brain and spinal cord, including Alzheimer’s disease (AD), Parkinson’s disease (PD), multiple sclerosis (MS), amyotrophic lateral sclerosis (ALS), and Huntington’s disease (HD) (Wilson et al., 2023). In recent years, the global prevalence of NDDs has steadily increased, with an alarming trend toward earlier onset in younger populations. This has profoundly impacted patients’ physical and mental well-being, significantly burdening public health systems and medical infrastructures (Bhat and Dhaneshwar, 2024). As a major contributor to global mortality and disability, NDDs are associated with diverse pathological mechanisms, including neuroinflammation, oxidative stress, mitochondrial dysfunction, synaptic impairment, and neuronal loss (Lv et al., 2025). Despite extensive research, targeted and effective therapies remain scarce. Most current therapeutic candidates for NDDs face challenges in crossing the blood–brain barrier, substantially limiting their clinical efficacy (Ross and Poirier, 2004). Moreover, patient compliance and interindividual variability further complicate precision medicine approaches for NDDs (Witzel et al., 2022; Malek and Grosset, 2015). Therefore, the in-depth investigation of novel therapeutic targets and alternative interventions holds significant theoretical importance and clinical value for the prevention and treatment of NDDs.

Iron is an essential trace metal required for the maintenance of normal physiological functions in humans. Iron metabolism encompasses critical processes—including absorption, transport, storage, utilization, and excretion—and plays a central role in erythropoiesis, oxygen transport, and the regulation of cellular respiration (Boyd et al., 2024). Ferroptosis is an iron-dependent form of regulated cell death, closely associated with disruptions in iron homeostasis, dysfunction of amino acid antioxidant systems, and lipid peroxidation (Jiang et al., 2021; Ryan et al., 2023a). A growing body of evidence has established ferroptosis as a critical driver in the initiation and progression of NDDs. Stimulation with 6-hydroxydopamine (6-OHDA) markedly downregulates nuclear factor erythroid 2–related factor 2 (NRF2) expression in PC-12 cells, elevates Fe^2+^ and reactive oxygen species (ROS) levels, suppresses glutathione peroxidase 4 (GPX4), promotes both ferroptosis and apoptosis, inhibits cell proliferation, and causes neuronal damage. Conversely, ferroptosis inhibitors significantly attenuate 6-OHDA-induced neuronal damage (Chen et al, 2025c). These findings suggest that targeted modulation of ferroptosis may represent a promising strategy for the prevention and treatment of NDDs. Exercise, as a critical adjunct to pharmacological therapy, has been extensively shown to confer multiple therapeutic benefits in NDDs, including suppression of neuronal ferroptosis, mitigation of neuroinflammation, reduction of Tau hyperphosphorylation, and enhancement of cognitive performance (Gutierre et al., 2025). Evidence has shown that 8 weeks of aerobic exercise significantly activates the System Xc^−^/GPX4 signaling axis in the prefrontal cortex of AD mice, upregulates ferritin light chain (FTL) and β-site amyloid precursor protein cleaving enzyme 1 (BACE1), downregulates 4-hydroxynonenal (4-HNE), inhibits ferroptosis and lipid peroxidation, and ameliorates cognitive deficits (Li et al., 2024a). However, the underlying mechanisms remain to be fully elucidated. On this basis, this review comprehensively examines the role of ferroptosis in NDDs and explores the mechanisms through which exercise modulates ferroptosis to alleviate NDD pathology, thereby providing a scientific foundation for exercise-based brain health interventions.

## 2 Ferroptosis overview

In 2003, Dolma and colleagues identified a novel compound, Erastin, through synthetic lethality screening designed to discover anticancer agents. Erastin selectively induces a non-apoptotic form of cell death in genetically engineered tumor cells co-expressing small T antigen (ST) and rat sarcoma viral oncogene homolog valine 12 (RAS^V12^). ST is the small T antigen encoded by simian virus 40 (SV40), and RAS^V12^ is an oncogenic RAS variant carrying a point mutation at codon 12. The co-expression of ST and RAS^V12^ leads to the upregulation of topoisomerase I, thereby increasing cellular susceptibility to Erastin (Dolma et al., 2003). Subsequently, Yagoda and Yang’s groups demonstrated that iron chelators could inhibit this type of cell death, discovered that Ras-selective lethal 3 (RSL3) mimicked Erastin’s effects in RAS-mutant cells, and found that elevated levels of ROS and iron were closely associated with RAS signaling (Yagoda et al., 2007; Yang and Stockwell, 2008). In 2012, while investigating the mechanism of Erastin-induced cell death, Dixon and colleagues formally coined the term “ferroptosis” to describe this iron-dependent, lipid peroxidation-mediated form of regulated cell death (Dixon et al., 2012).

**FIGURE 1:**
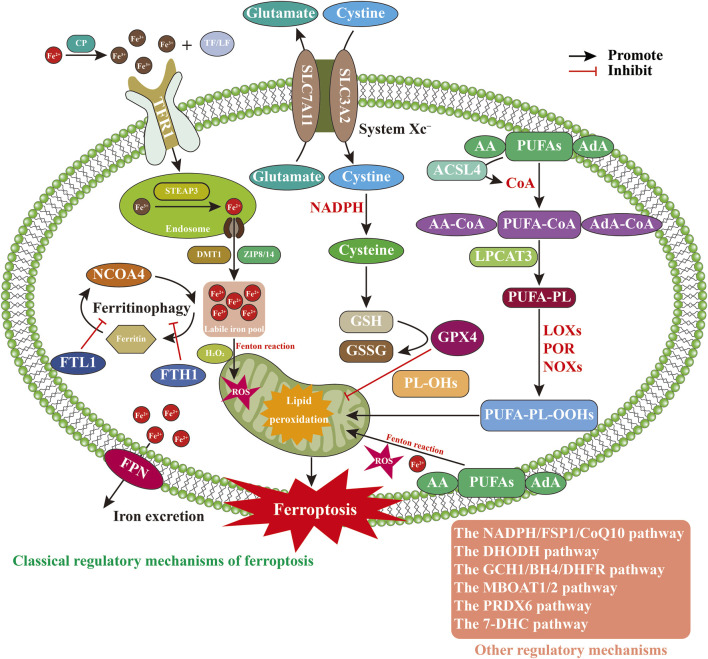
Regulatory mechanisms of ferroptosis. Abbreviations: 7-DHC, 7-dehydrocholesterol; AA, arachidonic acid; ACSL4, acyl-CoA synthetase long-chain family member 4; AdA, adrenic acid; BH4, tetrahydrobiopterin; CoA, coenzyme A; DHFR, dihydrofolate reductase; DHODH, dihydroorotate dehydrogenase; DMT1, divalent metal transporter 1; FPN, ferroportin; FSP1, ferroptosis suppressor protein 1; FTH1, ferritin heavy chain 1; FTL1, ferritin light chain 1; GCH1, GTP cyclohydrolase 1; GPX4, glutathione peroxidase; GSH, glutathione; LF, lactoferrin; LOXs, lipoxygenases; LPCAT3, lysophosphatidylcholine acyltransferase 3; MBOAT1/2, membrane-bound O-acyltransferase 1/2; NADPH, nicotinamideadenine dinucleotide phosphate; NCOA4, nuclear receptor coactivator 4; NOXs, NADPH oxidases; PL-OHs, phospholipid alcohols; POR, cytochrome P450 oxidoreductase; PRDX6, peroxiredoxin 6; PUFA-PL-OOHs, polyunsaturated fatty acid-containing phospholipid hydroperoxides; PUFA-PLs, polyunsaturated fatty acid–containing phospholipids; PUFAs, polyunsaturated fatty acids; ROS, reactive oxygen species; SLC3A2, solute carrier family 3 member 2; SLC7A11, solute carrier family 7 member 11; STEAP3, six-transmembrane epithelial antigen of prostate 3; TF, transferrin; TFR1, transferrin receptor protein 1.

Compared with other forms of cell death, such as autophagy, apoptosis, and pyroptosis, ferroptosis exhibits distinct morphological, biochemical, and immunological characteristics (Xia et al., 2025). Morphologically, ferroptosis is characterized by necrotic-like features, including loss of mitochondrial cristae, increased membrane density, outer membrane rupture, and reduced mitochondrial volume, while preserving plasma membrane integrity during early stages and maintaining normal nuclear morphology. Biochemically, ferroptosis involves iron dysregulation, ROS accumulation, mitochondrial dysfunction, glutathione (GSH) depletion, and lipid peroxidation (Liu et al., 2025). Immunologically, ferroptosis may trigger inflammatory responses and, in some cases, resemble inflammatory cell death (Zhu et al., 2025). The execution of ferroptosis involves complex regulation of multiple metabolic pathways (Figure 1), including disruptions in iron homeostasis, abnormalities in amino acid antioxidant systems, and the accumulation of lipid peroxides (Zheng et al., 2025; Costa et al., 2023).

### 2.1 Iron metabolism imbalance

Iron, an essential trace element, primarily participates in metabolic processes in the forms of Fe^2+^ and Fe^3+^ (Lee and Roh, 2025). Under physiological conditions, Fe^2+^ absorbed in the intestines or released from erythrocyte degradation is oxidized to Fe^3+^ by ceruloplasmin (CP) in the circulation. Fe^3+^ subsequently binds to transferrin (TF) or lactoferrin (LF) to form a TF–Fe^3+^ complex, which is then recognized and internalized by transferrin receptor 1 (TFR1) on the cell surface (Zhao et al., 2025b). Once inside the cell, Fe^3+^ is reduced to Fe^2+^ via six-transmembrane epithelial antigen of prostate 3 (STEAP3) and transported into the cytosol by divalent metal transporter 1 (DMT1) and ZRT/IRT-like proteins 8 and 14 (ZIP8/14) (Wang et al., 2025d). When iron stores are sufficient, most intracellular Fe^2+^ is sequestered in ferritin in an inactive form, while a small fraction forms the labile iron pool (LIP). Excess Fe^2+^ is exported via ferroportin (FPN) to maintain intracellular iron homeostasis (Wang et al., 2025d; Wang et al., 2025c). However, under conditions of iron overload, excess Fe^2+^ in the LIP undergoes Fenton chemistry with H_2_O_2_, producing excessive ROS, promoting lipid peroxidation, and ultimately triggering ferroptosis (Zhao et al., 2025a). Additionally, nuclear receptor coactivator 4 (NCOA4) binds to ferritin and promotes ferritinophagy, releasing labile Fe^2+^ into the LIP and facilitating ferroptosis. Conversely, upregulation of FTL1 and ferritin heavy chain (FTH1), or inhibition of NCOA4, can suppress ferroptosis (Feng et al., 2024b). In an *in vitro* model of Erastin-induced ferroptosis in PC12 neuronal cells, the iron inhibitor curcumin–polydopamine nanoparticles (Cur-PDA NPs) markedly enhanced the chelation of Fe^3+^ and Fe^2+^, upregulated superoxide dismutase (SOD) and GSH expression, reduced Fe^2+^ and ROS levels, inhibited lipid peroxidation, restored mitochondrial function, and attenuated neuronal ferroptosis. These results suggest that iron chelators can mitigate neuronal damage by limiting iron-induced oxidative stress and ferroptosis (Lei et al., 2024). However, iron chelators including deferoxamine (DFO), deferiprone (DFP), and deferasirox (DFX) generally lack tissue and cellular specificity, often resulting in the non-selective depletion of physiologically essential iron. This indiscriminate iron removal disrupts vital intracellular iron-dependent processes, such as mitochondrial respiration, DNA synthesis and repair, and redox reactions (Peña-Montes et al., 2024; Calabrese et al., 2020; Prabhune and Ameen, 2025). Furthermore, certain chelators can disrupt iron-dependent neurophysiological processes, thereby further constraining their clinical applicability in NDDs (Marupudi and Xiong, 2024). Therefore, iron metabolism imbalance, primarily driven by iron overload, is regarded as a critical driver of ferroptosis. While regulation of iron metabolism-related proteins or the use of iron chelators can reduce intracellular labile Fe^2+^ levels and inhibit ferroptosis, current chelators lack tissue and cellular specificity and may interfere with neurophysiological functions, thereby limiting their clinical applicability.

### 2.2 Abnormal amino acid antioxidant system

System Xc^−^, GSH, and GPX4 constitute a critical signaling axis in the regulation of intracellular lipid peroxidation and ferroptosis (Yu et al., 2025). System Xc^−^ is a membrane-bound amino acid antiporter composed of solute carrier family 7 member 11 (SLC7A11) and solute carrier family 3 member 2 (SLC3A2), connected via disulfide bonds. This transporter maintains the dynamic equilibrium between extracellular cystine and intracellular glutamate by importing cystine while exporting glutamate at a 1:1 ratio (Hu et al., 2025). Once inside the cell, cystine is reduced to cysteine through an NADPH-dependent process, serving as a key precursor for GSH synthesis and protecting against oxidative stress (Jiang and Sun, 2024). GSH, composed of glutamate, cysteine, and glycine, is a major intracellular antioxidant and exists in reduced GSH and oxidized glutathione (GSSG) forms (Yang et al., 2025). GPX4, a selenocysteine-containing enzyme, utilizes GSH to reduce toxic phospholipid hydroperoxides (PL-OOHs) to non-toxic phospholipid alcohols (PL-OHs), thereby disrupting lipid peroxidation chain reactions and preventing oxidative cell injury (Pontel et al., 2022). Dysfunction of System Xc^−^, depletion of GSH, or inhibition of GPX4 activity can lead to the accumulation of iron-dependent lipid peroxides, ultimately initiating ferroptosis (Guo et al., 2025a). Studies have confirmed that myricetin, a natural flavonoid, possesses multiple biological activities including antioxidant, anti-apoptotic, anti-inflammatory, and iron-chelating effects (Li et al., 2025a). Oral administration of myricetin significantly reduces Fe^2+^ levels in the substantia nigra of PD rats, inhibits neuronal ferroptosis, decreases ROS production, downregulates malondialdehyde (MDA) and α-synuclein (α-Syn) expression, elevates GSH levels, and ameliorates motor deficits. *In vitro*, myricetin treatment of SH-SY5Y neuroblastoma cells exposed to 1-methyl-4-phenylpyridine (MPP^+^) significantly reduces Fe^2+^ and ROS levels, activates the NRF2/GPX4 signaling pathway, suppresses neuronal ferroptosis, and mitigates cellular injury (Gu et al., 2024). Schisandrin B (Sch B), a natural lignan primarily derived from the traditional Chinese medicinal herb schisandra chinensis, exhibits anti-inflammatory, antioxidant, and neuroprotective properties (Dai et al., 2019). Administration of Sch B significantly inhibits glycogen synthase kinase 3β (GSK3β) activation in the hippocampus and cortex neurons of triple-transgenic Alzheimer’s disease (3 × Tg AD) mice, upregulates Nrf2, GPX4, and ferroptosis suppressor protein 1 (FSP1) expression, reduces levels of MDA, Fe^2+^, ROS, and tumor necrosis factor alpha (TNF-α), thereby suppressing neuronal ferroptosis and neuroinflammation and improving learning and memory performance. *In vitro*, Sch B suppresses erastin-induced ferroptosis in SH-SY5Y neuroblastoma cells by modulating the GSK3β/Nrf2/GPX4 signaling pathway (Ding et al., 2025). Moreover, radical-scavenging antioxidants such as Astragenol, Acteoside, and Edaravone have demonstrated neuroprotective effects in NDDs, primarily by neutralizing ROS, promoting GSH synthesis, and maintaining GPX4 activity to alleviate neuronal ferroptosis (Xiao et al., 2025; Wang et al., 2025a; Guo et al., 2023). However, the clinical efficacy of antioxidants remains controversial, mainly due to nonspecific mechanisms of action, insufficient target specificity, poor *in vivo* stability, and limited blood-brain barrier permeability (Ashok et al., 2022; Forman and Zhang, 2021). Therefore, GSH and GPX4, crucial regulators within the amino acid antioxidant system, play a critical role in inhibiting neuronal ferroptosis by maintaining their functional integrity. Although antioxidant therapies effectively inhibit ferroptosis, their clinical utility is limited by poor target specificity and instability. Thus, the development of highly specific drugs targeting key ferroptosis regulators is urgently needed.

### 2.3 Lipid peroxide accumulation

Polyunsaturated fatty acids (PUFAs), which are major constituents of organelle membrane phospholipids, are particularly prone to peroxidation during ferroptosis (Han et al., 2025). Among PUFAs, arachidonic acid (AA) and adrenic acid (AdA) are primary substrates for lipid peroxidation. Their activation and incorporation into phospholipids are orchestrated by acyl-CoA synthetase long-chain family member 4 (ACSL4) and lysophosphatidylcholine acyltransferase 3 (LPCAT3), two critical regulators of ferroptosis (Dixon et al., 2015; Poindessous et al., 2024). ACSL4 catalyzes the esterification of free AA and AdA with coenzyme A (CoA) to form PUFA-CoA derivatives (AA-CoA or AdA-CoA) (Doll et al., 2017). These activated fatty acyl-CoAs serve as acyl donors and are incorporated into lysophospholipids (Lyso-PLs), such as lysophosphatidylcholine (Lyso-PC) and lysophosphatidylethanolamine (Lyso-PE), by LPCAT3, yielding polyunsaturated fatty acid–containing phospholipids (PUFA-PLs) (Kagan et al., 2017; Liu et al., 2022a). Owing to their bis-allylic hydrogen atoms, PUFA-PLs are highly susceptible to peroxidation. This peroxidation occurs via two major pathways: the enzymatic pathway, mediated by lipoxygenases (LOXs), cytochrome P450 oxidoreductase (POR), and NADPH oxidases (NOXs), promotes the generation of PUFA-PL-OOHs, leading to the accumulation of reactive aldehydes, ketones, and other toxic metabolites, ultimately disrupting membrane integrity and triggering ferroptosis (Maheshwari, 2023); the non-enzymatic pathway involves ROS and Fe^2+^-driven Fenton reactions, which induce spontaneous oxidation of PUFA-PLs and further accumulation of PUFA-PL-OOHs (Kwun and Lee, 2023). Experimental evidence supports this mechanism. In a PD mouse model induced by intraperitoneal injection of salsolinol (SAL), and in SAL-treated SH-SY5Y cells, GSH expression was significantly reduced, whereas levels of ROS, Fe^2+^, ACSL4, MDA, 4-HNE, and α-Syn were markedly elevated. In addition, mitochondrial shrinkage and increased membrane density were observed, indicating that SAL induces ferroptosis and exacerbates neurotoxicity in both *in vivo* and *in vitro* PD models. Conversely, treatment with Ferrostatin-1 (Fer-1) or Acteoside, both ferroptosis inhibitors, significantly attenuated SAL-induced ferroptosis and neuronal injury (Wang et al., 2025b). These findings underscore lipid peroxide accumulation as a hallmark of ferroptosis, and suggest that downregulating ACSL4, LPCAT3, LOXs, or POR can suppress PUFA-PL-OOH formation and effectively inhibit ferroptosis.

### 2.4 Dysregulation of multi-control mechanisms

Ferroptosis is regulated by multiple parallel and complementary pathways (Zhao et al., 2025c; Long et al., 2024), including the NADPH/FSP1/CoQ10 pathway, the dihydroorotate dehydrogenase (DHODH) pathway, the GTP cyclohydrolase 1 (GCH1)/tetrahydrobiopterin (BH4)/dihydrofolate reductase (DHFR) pathway, the membrane-bound O-acyltransferase 1/2 (MBOAT1/2) pathway, the peroxiredoxin 6 (PRDX6) pathway, and the 7-dehydrocholesterol (7-DHC) pathway. NADPH, a major cellular reductant, supports the enzymatic activity of FSP1, which resides on the plasma and outer mitochondrial membranes. FSP1 catalyzes the reduction of CoQ10 to CoQ10H_2_, a lipophilic antioxidant that neutralizes PL-OOHs and halts lipid peroxidation, thereby suppressing ferroptosis independently of GPX4 (Doll et al., 2019; Bersuker et al., 2019; Chen et al., 2025b). DHODH, a mitochondrial inner membrane enzyme, catalyzes the oxidation of dihydroorotate (DHO) to orotate while transferring electrons to CoQ10, thereby generating CoQ10H_2_ that scavenges ROS and phospholipid hydroperoxides (PL-OOHs) within mitochondria and prevents ferroptosis (Mao et al., 2021; Xiang et al., 2024). The GCH1/BH4/DHFR axis functions independently of GPX4 and contributes to antioxidant defense through the synthesis of BH4. As a redox-active cofactor, BH4 directly scavenges PL-OOHs and indirectly promotes CoQ10 biosynthesis, thus conferring resistance to ferroptosis (Du et al., 2024). Upon oxidation, BH4 is converted to BH2, which is recycled back to BH4 by DHFR; inhibition of DHFR sensitizes cells to ferroptosis (Wang et al., 2024). MBOAT1 and MBOAT2, newly identified negative regulators of ferroptosis, remodel membrane phospholipids to reduce their susceptibility to oxidation. Their expression is transcriptionally controlled by estrogen receptor (ER) and androgen receptor (AR), respectively (Liang et al., 2023). PRDX6 is a multifunctional antioxidant enzyme that exhibits a broad spectrum of catalytic activities. Through its peroxidase activity, PRDX6 directly reduces PL-OOHs, providing antioxidant defense even in the absence of GPX4. Additionally, PRDX6 acts as a selenium-binding protein that promotes efficient intracellular selenium utilization, thereby sustaining GPX4 expression and activity and indirectly enhancing resistance to ferroptosis (Ito et al., 2024). 7-DHC functions as an intrinsic inhibitor of ferroptosis by incorporating into cellular membranes and selectively neutralizing lipid peroxyl radicals via the conjugated double bonds in its B-ring. Through sacrificial oxidation, 7-DHC produces protective derivatives, such as 3β,5α-dihydroxycholest-7-en-6-one (DHCEO), which disrupt lipid peroxidation chain reactions and mitigate ferroptosis (Freitas et al., 2024; Li et al., 2024b). Collectively, these parallel systems constitute a multi-tiered defense network that maintains redox balance and modulates ferroptosis sensitivity.

## 3 Ferroptosis and neurodegenerative diseases

Iron is an essential trace element for maintaining brain function, playing vital roles in neuronal development, neurotransmitter synthesis, mitochondrial energy metabolism, myelin formation, and synaptic plasticity (Gao et al., 2025). However, disruptions in iron homeostasis, coupled with impaired amino acid antioxidant systems, can markedly deplete intracellular GSH in neurons. This exacerbates lipid peroxidation, drives excessive ROS accumulation and neuroinflammation, and ultimately triggers ferroptosis, resulting in irreversible neuronal damage and neurotoxicity (Chen et al., 2025a). Increasing evidence highlights ferroptosis as a pivotal contributor to the pathogenesis of NDDs (Table 1), suggesting that therapeutic targeting of ferroptosis may offer a promising strategy for the prevention and treatment of NDDs.

**TABLE 1:** Role of ferroptosis in neurodegenerative diseases.

NDDs	Compounds/Drugs/Proteins/RNA	Ferroptosis	Marker proteins	Main functions	Refs
AD	Ghrelin ↑	↓	BMP6, SMAD1, SLC7A11, GPX4, FTL1, FTH1, Arg-1, IL-10, TGF-β ↑	Promoted the polarization of microglia towards M2, and improved learning and memory impairments in AD mice	Guo et al. (2025b)
	Neuritin ↑	↓	Map2, NeuN, PSD95, GSH, NADPH ↑ ROS, MDA, 4-HNE ↓	Enhanced neuronal signal transmission and synaptic plasticity, inhibited neuronal oxidative stress, and improved cognitive impairment and learning and memory abilities in AD mice	Song et al. (2025)
	GAA ↑	↓	GPX4, SLC7A11, NRF2, FTH1, SOD, GSH ↑ Fe^2+^, TFR1, ACSL4, MDA, GSSG ↓	Improved the learning and memory abilities of AD mice	Lu et al. (2025)
	Artemisinin ↑	↓	NRF2, SLC7A11, GPX4, GSH ↑ KEAP1, MDA, ROS ↓	Improved the learning and memory abilities of AD mice	Deng et al., (2025)
PD	Neural stem cell-derived exosomes CDC42 ↑	↓	GSH, GPX4, VEGF, IL-8 ↑ ACSL4, ROS, MDA, 4-HNE, Fe^2+^, α-Syn ↓	Reduced cerebral vascular damage and cognitive and memory dysfunction in PD mice	Li et al. (2025b)
	TIGAR ↑	↓	NADPH, GPX4, GSH, ↑ GSSG, Fe^2+^, MDA, ROS ↓	Inhibited ferroptosis in dopaminergic neurons and improved neurological function	Sheng et al. (2025)
	FTO ↓	↓	YTHDF2, SLC7A11 ↑ BAP1, α-Syn, MDA, Fe^2+^, 4-HNE ↓	Improved the health of dopaminergic neurons in PD mice	Li et al. (2025c)
	IRP2 ↓	↓	SLC7A11, GPX4 ↑ p53, TFR1, FTH, ALOX12 ↓	Reduced PC-12 cell damage	Yao et al. (2024)
MS	MAT ↑	↓	GSH, SOD, GPX4, SLC7A11 ↑ MDA, LPCAT3, PTGS2, IL-6, TNF-α, IL-1β ↓	Reduced neuroinflammation and central nervous system damage in EAE mice	Feng et al. (2024a)
	Dabrafenib ↑	↓	Axl, System Xc^−^, GPX4, Ferritin ↑ CD71, ACSL4, POR, Iba-1, ROS ↓	Improved gait abnormalities and limb weakness in EAE mice	Liu et al. (2024)
	Bone marrow mesenchymal stem cell-derived exosomes miR-367-3p ↑	↓	SLC7A11, GPX4, GSH, SOD↑ EZH2, Fe^2+^, MDA, ↓	Reduced inflammation and injury to the spinal cord	Fan et al. (2023)
	DFP ↑	↓	GSH, NRF2, GPX4 ↑ Fe^2+^, TFR1, Iba-1 ↓	Reduced demyelination and optic nerve damage in mice	Rayatpour et al. (2022)
ALS	25-OHC ↑	↑	PTGS2, ROS, CYB5R1, POR ↑ SREBP, GXP4, SCD1, GSH ↓	Aggravated damage to glial cells	Urano et al. (2025)
	NRF2 ↓	↑	MDA, GSH, ROS ↑ SLC7A11, GPX4 ↓	Aggravated motor neuron damage	Yang et al. (2023)
	SPY1 ↓	↑	ALOX15, GDF15, TFR1, Fe^2+^ ↑ GCH1, GPX4, GSH ↓	Exacerbated muscle atrophy and motor dysfunction in mice	Wang et al. (2023)
	MPO ↑	↑	HOCl, Caspase-3, MDA ↑ GPX4, NQO1 ↓	Reduces motor performance in mice	Peng et al. (2022)

Abbreviations: BMP6, bone morphogenetic protein 6; SLC7A11, solute carrier family 7 member 11; GPX4, glutathione peroxidase; FTL1, ferritin light chain; FTH1, ferritin heavy chain 1; Arg-1, arginase 1; IL-10, lnterleukin-10; TGF-β, transforming growth factor-β; Map2, microtubule-associated protein 2; NeuN, neuronal nuclei; PSD95, postsynaptic density protein 95; GSH, glutathione; NADPH, nicotinamideadenine dinucleotide phosphate; ROS, reactive oxygen species; MDA, malondialdehyde; GAA, ganoderic acid A; 4-HNE, 4-hydroxynonenal; NRF2, nuclear factor-erythroid 2 related factor 2; SOD, superoxide dismutase; TFR1, transferrin receptor protein 1; ACSL4, acyl-CoA, synthetase long-chain family member 4; GSSG, oxidized glutathione; KEAP1, kelch-like ECH-associated protein 1; VEGF, vascular endothelial growth factor; TIGAR, Tp53-induced glycolysis and apoptosis regulator; α-Syn, α-Synucleinα; FTO, fat mass and obesity-associated protein; YTHDF2, YTH, domain family protein 2; BAP1, BRCA1-associated protein 1; IRP2, iron regulatory protein 2; ALOX12, arachidonate 12-Lipoxygenase; MAT, matrine; LPCAT3, lysophosphatidylcholine acyltransferase 3; PTGS2, prostaglandin-endoperoxide synthase 2; TNF-α, tumor necrosis factor alpha; EAE, experimental autoimmune encephalomyelitis; Axl, Axl receptor tyrosine kinase; POR, cytochrome P450 oxidoreductase; Iba-1, ionized calcium-binding adapter molecule 1; EZH2, enhancer of zeste homolog 2; DFP, deferiprone; 25-OHC, 25-hydroxycholesterol; CYB5R1, cytochrome b5 reductase 1; SREBP, sterol regulatory element-binding protein; SCD1, stearoyl-CoA, desaturase 1; SPY1, speedy/RINGO, cell cycle regulator family member A; GDF15, growth differentiation factor 15; GCH1, GTP, cyclohydrolase 1; MPO, myeloperoxidase; HOCl, hypochlorous acid; Caspase-3, cystein-asparate protease 3; NQO1, quinone oxidoreductase 1; NDDs, neurodegenerative diseases; Refs, References; AD, alzheimer’s disease; PD, parkinson’s disease; MS, multiple sclerosis; ALS, amyotrophic lateral sclerosis.

### 3.1 Clinical studies of ferroptosis in neurodegenerative diseases

Human biospecimen analyses revealed elevated agrin expression, reduced levels of platelet-derived growth factor receptor beta (PDGFRβ), type IV collagen, and fibronectin, pronounced small artery atherosclerosis and venous collagen deposition, and a reduction in pericyte numbers in the brain tissue of AD patients. Notably, basement membrane–associated extracellular matrix remodeling was closely associated with neuropathological alterations in AD. *In vitro* experiments demonstrated that agrin treatment significantly downregulated PDGFRβ and claudin-5 expression in a blood-brain barrier co-culture model comprising human brain microvascular endothelial cells and pericytes, suggesting that agrin may induce pericyte death and impair the integrity of the blood-brain barrier. Moreover, agrin-treated pericytes exhibited elevated levels of ROS and Fe^2+^, along with reduced expression of GPX4 and FTH1, thereby promoting ferroptosis, suppressing pericyte proliferation, and compromising blood-brain barrier function. Notably, treatment with Fer-1, a ferroptosis inhibitor, significantly alleviated pericyte injury (Cao et al., 2025). In a co-culture model comprising neurons, astrocytes, and microglia derived from human induced pluripotent stem cells, exposure to iron and the ferroptosis inducer RSL3 demonstrated that microglia were the most susceptible to iron overload. FTH1 and lnterleukin-8 (IL-8) expression was markedly increased in microglia. Neurons showed reduced cell surface area, elevated lipid peroxidation, and characteristic features of ferroptosis. Notably, microglia removal significantly mitigated ferroptosis. Consistently, *postmortem* striatal brain tissue from three patients with PD exhibited elevated FTH1 and IL-8 expression, closely recapitulating the transcriptional profile of microglia in the *in vitro* model. Furthermore, genome-wide CRISPR screening identified SEC24 homolog B (SEC24B) as a key regulator of ferroptosis, and its knockout significantly inhibited ferroptosis in microglia (Ryan et al., 2023b). Experimental evidence demonstrated that stromal interaction molecule 1 (STIM1) expression was significantly downregulated in cortical neurons of mice with experimental autoimmune encephalomyelitis (EAE) induced by MOG35-55. This downregulation facilitated the dissociation of STIM1 from STING, thereby activating the non-canonical STING signaling pathway and enhancing autophagy in cortical neurons. Increased colocalization of GPX4 with the autophagy marker microtubule-associated protein 1 light chain 3 (LC3) promoted autophagic degradation of GPX4. As a result, GPX4 and GSH levels were markedly reduced, accompanied by elevated ROS levels and increased lipid peroxidation, collectively driving ferroptosis in cortical neurons and leading to neurodegeneration. Neuron-specific STING knockout significantly attenuated neuronal damage in EAE mice. Notably, a similar phenomenon was observed in neurons from patients with MS, characterized by upregulated STING expression and markedly reduced STIM1 expression, suggesting that STIM1-STING-GPX4 axis-mediated ferroptosis is closely associated with neuronal injury in human MS pathology (Woo et al., 2024). In spinal cord tissues from patients with ALS, the expression of arachidonate 5-Lipoxygenase (ALOX5) and complement component 3 (C3) was markedly upregulated, accompanied by reduced GSH levels, elevated iron concentrations, and enhanced lipid peroxidation. Microglia in ALS spinal cords exhibited increased expression of ALOX5 and LPCAT3, indicating dysregulated iron metabolism and activation of ferroptosis. These alterations suggest that ferroptosis may contribute to the activation of neurotoxic glial cells and exacerbate neuronal damage in ALS. In a co-culture system of microglia, astrocytes, and neurons exposed to RSL3, ALOX5 was predominantly enriched in microglia. RSL3 stimulation elevated C3, IL-1α, and TNF-α levels in mixed glial cultures, while the ferroptosis inhibitor liproxstatin-1 effectively attenuated RSL3-induced neurotoxicity, supporting a role for ferroptotic stress in promoting neurotoxic glial activation. Consistently, SOD1^G93A^ transgenic mice exhibited ferroptotic features and neurotoxic glial activation resembling those observed in ALS patient spinal cords and *in vitro* models. Remarkably, treatment with CuII(atsm) significantly mitigated neuronal injury in the ALS mouse model (Liddell et al., 2024).

In summary, ferroptosis is intimately linked to the initiation and progression of AD, PD, MS, and ALS, as evidenced by strong concordance between findings from clinical human samples and those derived from animal and *in vitro* models. However, the majority of current clinical studies are still based on isolated case reports or small cohorts, limiting the ability to systematically characterize the prevalence and disease-stage- and population-specific features of ferroptosis.

### 3.2 Preclinical studies of ferroptosis in neurodegenerative diseases

#### 3.2.1 Ferroptosis and alzheimer’s disease

AD is a prevalent and progressive neurodegenerative disorder, characterized by the accumulation of extracellular β-amyloid (Aβ) plaques and intracellular neurofibrillary tangles formed by hyperphosphorylated Tau protein, accompanied by excessive neuroinflammation, synaptic dysfunction, and neuronal loss, ultimately resulting in impairments in language, judgment, and executive function, and even leading to the loss of independent living ability (Hooper et al., 2025). Emerging evidence indicates that growth hormone-releasing peptide Ghrelin and the neurotrophic factor Neuritin suppress neuronal ferroptosis and lipid peroxidation, thereby mitigating brain injury and improving cognitive function. Aβ_1-42_ stimulation markedly suppressed the activation of the bone morphogenetic protein 6 (BMP6)/SMAD1 signaling pathway in BV2 microglial cells, resulting in the downregulation of SLC7A11, GPX4, FTL1, and FTH1 expression, increased ROS and MDA levels, and decreased GSH and SOD levels. This was accompanied by upregulation of inducible nitric oxide synthase (iNOS), IL-1β, and TNF-α, and downregulation of arginase 1 (Arg-1), IL-10, and transforming growth factor-beta (TGF-β). These changes induced ferroptosis, mitochondrial damage, and neuroinflammation in BV2 cells. In contrast, Ghrelin treatment activated the BMP6/SMAD1 signaling pathway, which upregulated the expression of SLC7A11, GPX4, FTL1, and FTH1. It also promoted the expression of Arg-1, IL-10, and TGF-β, inhibiting ferroptosis and inducing BV2 cell polarization toward the M2 phenotype, thereby mitigating BV2 cell injury. *In vivo* studies confirmed that Ghrelin significantly activated the BMP6/SMAD1 signaling pathway in the brain tissue of AD mice, thereby inhibiting microglial ferroptosis, promoting microglial polarization toward the M2 phenotype, and ameliorating learning and memory deficits in AD mice (Guo et al., 2025b). Neuritin significantly upregulated the expression of microtubule-associated protein 2 (Map2), neuronal nuclei (NeuN), and postsynaptic density protein 95 (PSD95) in the hippocampus of Amyloid precursor protein (APP)/Presenilin 1 (PS1) transgenic AD mice. It also reduced the accumulation of ROS, MDA, and 4-HNE, while enhancing GSH and NADPH expression and the NADP/NADPH ratio. These effects improved neuronal signaling and synaptic plasticity, inhibited neuronal ferroptosis and oxidative stress, and alleviated cognitive deficits, as well as enhanced learning and memory in AD mice. *In vitro*, Neuritin activated the phosphoinositide 3-kinase (PI3K)/protein kinase B (Akt) signaling pathway in Aβ_1-42_-stimulated HT22 neuronal cells, leading to increased NADK activity and NADPH expression, reduced MDA and ROS levels, and inhibition of neuronal ferroptosis. The ferroptosis activator RSL-3 and the PI3K/Akt pathway inhibitor LY294002 reversed Neuritin’s inhibitory effect on ferroptosis, indicating that Neuritin suppresses neuronal ferroptosis via PI3K/Akt pathway activation (Song et al., 2025). Additional studies have demonstrated that ganoderic acid A (GAA) and artemisinin may mitigate neuronal damage by modulating iron metabolism and the antioxidant system. GAA is an aldosterone-type triterpenoid derived from Ganoderma lucidum, known for its antioxidant, anti-inflammatory, antidepressant, and anticancer properties (Chen X et al., 2024). GAA administration significantly upregulated the expression of GPX4, SLC7A11, and NRF2 in the hippocampus of APP/PS1 transgenic AD mice, inhibited neuronal ferroptosis, and improved cognitive function in these mice. *In vitro* experiments confirmed that Aβ_25-35_ treatment significantly inhibited the activation of the NRF2/SLC7A11/GPX4 signaling pathway in HT22 cells, upregulated the expression of Fe^2+^, TFR1, ACSL4, MDA, and GSSG, and decreased the expression of FTH1, SOD, and GSH, as well as the GSH/GSSG ratio, resulting in dysregulated neuronal iron metabolism, impaired amino acid antioxidant systems, mitochondrial dysfunction, and ferroptosis, which ultimately promoted neuronal injury. In contrast, GAA and the ferroptosis inhibitor liproxstatin-1 (Lip-1) significantly activated the NRF2/SLC7A11/GPX4 signaling pathway and mitigated Aβ_25-35_-induced neuronal injury (Lu et al., 2025). Artemisinin, a herbal extract from Artemisia annua, possesses anti-inflammatory, antitumor, and antiviral properties (Tu, 2016). Cellular assays revealed that Artemisinin binds competitively to Kelch-like ECH-associated protein 1 (KEAP1), inhibiting its expression in Aβ_1-42_-stimulated HT22 cells. This interaction promotes the dissociation of the KEAP1-NRF2 complex, preventing NRF2 ubiquitination and degradation, thereby enhancing NRF2 expression. Activation of the NRF2/SLC7A11/GPX4 signaling pathway leads to elevated GSH levels, reduced MDA and ROS accumulation, suppression of neuronal ferroptosis, lipid peroxidation, and amino acid antioxidant dysfunction, ultimately mitigating neuronal injury. *In vivo*, Artemisinin significantly decreased KEAP1 expression while increasing NRF2 levels in the hippocampus of 3 × Tg AD mice. This activation of the NRF2/SLC7A11/GPX4 pathway enhanced GSH levels, decreased lipid ROS accumulation, inhibited ferroptosis and lipid peroxidation, and improved the cognitive functions of AD mice (Deng et al., 2025).

In summary, Ghrelin, Neuritin, GAA, and Artemisinin may mitigate cerebral neuronal injury and cognitive dysfunction by modulating neuronal ferroptosis, lipid peroxidation, the antioxidant system, oxidative stress, mitochondrial dysfunction, and neuroinflammation. However, existing studies primarily focus on short-term intervention effects at the cellular and animal levels, with a notable absence of comprehensive long-term behavioral assessments and safety evaluations. Consequently, the clinical applications of these interventions warrant further investigation.

#### 3.2.2 Ferroptosis and parkinson’s disease

PD is the second most prevalent NDD, characterized by the degeneration and loss of dopaminergic neurons in the substantia nigra of the midbrain. This pathology is accompanied by the accumulation of misfolded α-Syn, forming eosinophilic inclusion bodies known as Lewy bodies, alongside neuroinflammation. Clinically, PD presents with dyskinesia, bradykinesia, resting tremor, postural instability, and rigidity (Rosal et al., 2025). Several studies have shown that overexpression of CDC42 from neural stem cell-derived exosomes and Tp53-induced glycolysis and apoptosis regulator (TIGAR) significantly suppresses lipid peroxidation and ferroptosis, thereby promoting neurological recovery. Hypoxia-preconditioned neural stem cell-derived exosomes enriched in CDC42 significantly downregulate ACSL4 expression in MPP^+^-stimulated human cerebral microvascular endothelial cells (HCMECs). They simultaneously elevate GSH and GPX4 levels, reduce the accumulation of ROS, MDA, 4-HNE, and Fe^2+^, and upregulate vascular endothelial growth factor (VEGF) and IL-8 expression. These exosomes inhibit MPP^+^-induced ferroptosis and lipid peroxidation, enhance HCMEC viability, wound healing, and migratory capacity, and promote cerebral angiogenesis. *In vivo*, 1-methyl-4-phenyl-1,2,3,6-tetrahydropyridine (MPTP)-induced PD mice exhibited a marked reduction in tyrosine hydroxylase (TH)-positive neurons and CD31 expression, along with significant decreases in GSH and GPX4 levels. Conversely, ACSL4 expression, along with levels of ROS, MDA, 4-HNE, Fe^2+^, and α-synuclein, were markedly elevated, thereby exacerbating ferroptosis and lipid peroxidation in the brain. Treatment with Fer-1, Lip-1, or neural stem cell-derived extracellular vesicles enriched in CDC42 substantially attenuates cerebrovascular damage and cognitive deficits in PD mice (Li et al., 2025b). MPP^+^ treatment significantly reduces TIGAR expression in HT22 cells, downregulates NADPH, GPX4, and GSH levels, upregulates GSSG levels and the GSSG/GSH ratio, decreases adenosine triphosphate (ATP) production, and promotes neuronal ferroptosis, iron-dependent lipid peroxidation, and mitochondrial dysfunction, thereby exacerbating neuronal cytotoxicity. In contrast, TIGAR overexpression or treatment with Fer-1, a ferroptosis inhibitor, significantly increases NADPH, GPX4, and GSH levels, reduces GSSG expression, and attenuates neuronal ferroptosis and neurological impairment. *In vivo*, TIGAR overexpression markedly increases NADPH, GPX4, and GSH levels in the substantia nigra of MPTP-induced PD mice, reduces Fe^2+^, MDA, and ROS levels, inhibits dopaminergic neuronal ferroptosis, and improves neurological function (Sheng et al., 2025). Several studies have demonstrated that silencing fat mass and obesity-associated protein (FTO) and iron regulatory protein 2 (IRP2) mitigates abnormalities in the amino acid antioxidant system and ferroptosis, thereby attenuating dopaminergic neuronal damage. N6-methyladenosine (m^6^A) demethylase FTO is significantly upregulated in MPP^+^-stimulated SK-N-SH cells, which suppresses YTH domain family protein 2 (YTHDF2) expression, promotes m^6^A demethylation of BRCA1-associated protein 1 (BAP1) and enhances BAP1 expression, upregulates p53 expression, and inhibits SLC7A11 transcription, thereby increasing MDA, Fe^2+^, and 4-HNE levels and triggering neuronal ferroptosis. *In vivo*, FTO knockdown significantly elevates YTHDF2 expression in the striatum of PD mice, inhibits m^6^A demethylation of BAP1 and reduces BAP1 expression, upregulates SLC7A11 expression, decreases α-Syn, MDA, Fe^2+^, and 4-HNE levels, suppresses neuronal ferroptosis and disruptions in the amino acid antioxidant system, and promotes dopaminergic neuronal survival in PD mice (Li et al., 2025c). IRP2 expression is significantly upregulated in the midbrain substantia nigra of PD mice, increasing TFR1, FTH, and p53 levels, while decreasing SLC7A11 and GPX4 levels, leading to ferroptosis and disruption of the amino acid antioxidant system in dopaminergic neurons. This results in a reduction in the number of TH-positive neurons and dopamine content, thereby exacerbating dopaminergic neuronal damage in PD mice. Overexpression of IRP2 or MPP^+^ treatment significantly enhances the binding of IRP2 to p53 in PC-12 cells, upregulates p53 levels, downregulates SLC7A11 expression, and increases arachidonate 12-Lipoxygenase (ALOX12) expression, thereby promoting ferroptosis and amino acid antioxidant system disruptions, elevating MDA levels, and inducing PC-12 cell damage. In contrast, IRP2 knockdown or treatment with the ferroptosis inhibitor Fer-1 alleviates PC-12 cell damage by downregulating p53 levels and activating the SLC7A11-ALOX12 signaling pathway (Yao et al., 2024).

In summary, CDC42, derived from neural stem cell-derived exosomes, as well as TIGAR, FTO, and IRP2, contribute to PD pathology by regulating iron homeostasis, ferroptosis, fatty acid metabolism, the antioxidant system, and angiogenesis. However, most published studies primarily focus on the interrelationships between ferroptosis, iron metabolism, lipid peroxidation, and amino acid antioxidant systems, with limited exploration into the interconnected roles of ferroptosis and other mechanisms, such as mitochondrial quality control, neuroinflammation, and neurogenesis.

#### 3.2.3 Ferroptosis and multiple sclerosis

MS is an autoimmune-mediated NDD characterized by abnormal activation of immune cells, leading to myelin loss, axonal degeneration, and astrocyte proliferation. This is accompanied by mitochondrial dysfunction, oxidative stress, and neuroinflammation, ultimately triggering neuronal decline (Li et al., 2024c). Patients with MS often exhibit symptoms such as motor deficits, muscle weakness, sensory abnormalities, cognitive decline, and psychiatric abnormalities, which significantly affect the quality of life (Sturm et al., 2014). Studies have confirmed that matrine (MAT) and Dabrafenib inhibit ferroptosis and neuroinflammation, thereby alleviating neurological dysfunction. MAT is a quinolizidine-rich natural alkaloid with diverse biological effects, including anti-inflammatory, antifibrotic, antiviral, and analgesic properties (Jin et al., 2024). Animal studies revealed that the expression of GSH, SOD, GPX4, and SLC7A11 was significantly downregulated in the cerebrospinal fluid of EAE mice induced by MOG35-55 peptide injection. MDA, LPCAT3, and prostaglandin-endoperoxide synthase 2 (PTGS2) expression were significantly increased, promoting neuronal ferroptosis and lipid peroxidation in EAE mice. This also significantly elevated pro-inflammatory factors IL-6, TNF-α, and IL-1β in the cerebrospinal fluid, and upregulated lipoxygenase (LOX), PTGS2, IL-6, and TNF-α expression in microglial cells, which further promote inflammatory activation. In contrast, treatment with MAT significantly inhibits the inflammatory responses caused by ferroptosis and lipid peroxidation in the spinal cord, attenuating central nervous system damage in EAE mice (Feng et al., 2024a). Dabrafenib treatment significantly upregulated the expression of Axl receptor tyrosine kinase (Axl) in the spinal cord tissues of EAE mice, increased the expression of System Xc^−^, GPX4, and Ferritin, and decreased the expression of CD71, ACSL4, and POR. Additionally, it downregulated the expression of ionized calcium-binding adapter molecule 1 (Iba-1), inhibited spinal cord ferroptosis and inflammatory responses, and improved gait abnormalities and limb weakness in EAE mice. Furthermore, Axl knockdown significantly exacerbated spinal cord ferroptosis and the inflammatory response in EAE mice, suggesting that Axl may serve as a key target for regulating ferroptosis. Cell experiments showed that Dabrafenib significantly upregulated Axl expression in BV2 cells co-stimulated with lipopolysaccharide (LPS) and Erastin. It downregulated ACSL4 and CD71 expression, significantly increased the expression of System Xc^−^, GPX4, and Ferritin, reduced ROS levels, and inhibited ferroptosis, inflammatory responses, and mitochondrial dysfunction in BV2 cells. Additionally, Dabrafenib alleviated microglial inflammatory injury (Liu et al., 2024). Several studies have confirmed that microRNA-367-3p (miR-367-3p), derived from exosomes of bone marrow mesenchymal stem cells, and DFP inhibit iron deposition and ferroptosis, thereby promoting neurological recovery. Bone marrow mesenchymal stem cell-derived exosomes miR-367-3p, in Erastin-induced BV2 cells, targets the binding enhancer of zeste homolog 2 (EZH2), inhibiting its expression. This, in turn, upregulates SLC7A11 expression, enhances the levels of GPX4, GSH, and SOD, and reduces Fe^2+^ and MDA levels, thereby suppressing ferroptosis and promoting BV2 cell survival. Animal experiments confirmed that dural injection of bone marrow mesenchymal stem cell-derived exosomes miR-367-3p significantly reduced the expression of EZH2 in spinal cord tissues of EAE mice, increased the expression of SLC7A11, GPX4, GSH, and SOD, decreased the levels of Fe^2+^ and MDA, inhibited spinal cord iron deposition, ferroptosis, and inflammation, and alleviated spinal cord injury (Fan et al., 2023). Optic nerve injection of lysophosphatidylcholine (LPC) induced localized myelin loss in MS mice, significantly elevating Fe^2+^ and MDA levels, while reducing GSH, NRF2, and GPX4 expression, as well as iron reactive element binding protein 2 (IREB2) in optic nerve tissue. Additionally, TFR1, acyl-CoA synthetase family member 2 (ACSF2), and heme oxygenase-1 (HO-1) expression were significantly upregulated, leading to increased ferroptosis in the optic nerves of locally demyelinated MS mice. In contrast, DFP treatment significantly decreased Fe^2+^ levels in optic nerve tissue, downregulated TFR1 and Iba-1 expression, and inhibited iron deposition, ferroptosis, microglial activation, and astrocyte proliferation, thereby reducing myelin loss and optic nerve damage in MS mice (Rayatpour et al., 2022).

In summary, MAT, Dabrafenib, bone marrow mesenchymal stem cell-derived exosomes miR-367-3p, and DFP modulate ferroptosis, iron deposition, lipid peroxidation, neuroinflammation, microglial activation, and myelin loss, thereby attenuating MS-related pathological damage. However, most current studies rely solely on the EAE mouse model and the LPC-induced localized myelin loss model, which do not fully recapitulate the complex pathological features of MS, particularly its heterogeneity, disease progression, and clinical symptoms.

#### 3.2.4 Ferroptosis and amyotrophic lateral sclerosis

ALS is a lethal NDD characterized by progressive degeneration of upper and lower motor neurons in the cerebral cortex, brainstem, and spinal cord, leading to skeletal muscle atrophy, weakness, weight loss, dysarthria, dysphagia, paralysis, and ultimately death from respiratory failure (Feldman et al., 2022). Studies have confirmed that 25-hydroxycholesterol (25-OHC) promotes glial ferroptosis and lipid peroxidation, thereby contributing to ALS progression. 25-OHC treatment significantly inhibited sterol regulatory element-binding protein (SREBP) expression in IMS32 Xuewang cells, downregulated GXP4 and stearoyl-CoA desaturase 1 (SCD1), and upregulated PTGS2, thereby increasing intracellular ROS levels and promoting ferroptosis. Meanwhile, 25-OHC markedly upregulated cytochrome b5 reductase 1 (CYB5R1) and POR, suppressed GSH expression, and promoted lipid peroxidation, thereby enhancing the sensitivity of IMS32 cells to ferroptosis and aggravating neuroglial injury. Inhibition of 25-OHC expression or treatment with Fer-1, a ferroptosis inhibitor, significantly suppressed ferroptosis and lipid peroxidation in IMS32 cells and alleviated neurological damage, indicating that 25-OHC may serve as a potential therapeutic target for ALS (Urano et al., 2025). Other studies have confirmed that NRF2, speedy/RINGO cell cycle regulator family member A (SPY1), and myeloperoxidase (MPO) regulate ferroptosis and lipid peroxidation in motor neurons, thereby modulating neural function. NRF2 expression was markedly decreased in NSC-34 motor neuron cells transfected with the human SOD1^G93A^ gene, leading to significantly increased levels of MDA, GSH, and ROS, along with reduced expression of SLC7A11 and GPX4. These changes promoted ferroptosis and lipid peroxidation in NSC-34 cells, decreased cell viability, and aggravated motor neuron damage. *In vivo*, intraperitoneal administration of the NRF2 activator RTA-408 significantly upregulated NRF2 expression in neurons of SOD1^G93A^ transgenic mice, which consequently elevated the expression of GSH, SLC7A11, and GPX4, reduced MDA and ROS levels, suppressed neuronal ferroptosis, lipid peroxidation, and mitochondrial injury, and ameliorated weight loss and motor dysfunction in mice (Yang et al., 2023). The level of murine double minute 2 (MDM2) was significantly upregulated in neurons of SOD1^G93A^ transgenic mice. MDM2 mediates the ubiquitin-dependent degradation of SPY1, resulting in a downregulation of SPY1 expression, which in turn upregulates the expression of ALOX15 and growth differentiation factor 15 (GDF15), while decreasing the levels of GCH1, GPX4, and GSH. This cascade leads to a significant increase in TFR1 and Fe^2+^ levels, promoting neuronal ferroptosis and lipid peroxidation, which exacerbates muscle atrophy and motor dysfunction in mice. *In vitro* experiments confirmed that SPY1 overexpression significantly activated the GCH1/BH4 signaling pathway in NSC-34 cells transfected with the human SOD1^G93A^ gene. It reduced TFR1, p53, and Fe^2+^ levels, increased GSH expression, inhibited ferroptosis and lipid peroxidation, and ameliorated neuronal injury (Wang et al., 2023). MPO and hypochlorous acid (HOCl) expression were significantly upregulated in NSC-34 cells transfected with the human SOD1^G93A^ gene, leading to the activation of the MPO/HOCl signaling pathway. This activation significantly increased the Bcl-2-associated X protein (Bax)/B-cell lymphoma 2 (Bcl-2) ratio and cysteine-aspartate protease 3 (Caspase-3) expression, inhibited GPX4 and quinone oxidoreductase 1 (NQO1) expression, increased MDA levels, and promoted apoptosis, ferroptosis, and lipid peroxidation in NSC-34 cells. Overexpression of FSP1 or treatment with Fer-1, a ferroptosis inhibitor, significantly reversed the apoptosis, ferroptosis, and lipid peroxidation induced by the activation of the MPO/HOCl signaling pathway, exerting neuroprotective effects. *In vivo* experiments confirmed that the MPO/HOCl signaling pathway was significantly activated in the plasma and neurons of SOD1^G93A^ transgenic mice, which upregulated Caspase-3 expression, downregulated GPX4 and NQO1 expression, significantly elevated plasma MDA levels, promoted neuronal apoptosis, ferroptosis, and lipid peroxidation, and reduced motor performance in mice (Peng et al., 2022).

In summary, 25-OHC, NRF2, SPY1, and MPO regulate neuronal ferroptosis and lipid peroxidation, thereby participating in the pathological development of ALS. However, the potential side effects of NRF2 activators and ferroptosis inhibitors have not been thoroughly evaluated. Specifically, excessive activation of NRF2 may promote cell proliferation and tumorigenesis, highlighting the need for a comprehensive assessment of safety and side effects before clinical application.

## 4 Clinical studies of exercise interventions in neurodegenerative diseases

Exercise, as a low-cost and highly accessible non-pharmacological intervention, has received growing attention for the prevention and treatment of NDDs (Sanaeifar et al., 2024). A growing body of clinical research indicates that exercise interventions can markedly enhance cognitive performance, motor function, and overall quality of life in individuals with NDDs (Santamaría et al., 2025). A forty-minute cycling training protocol significantly increased cortical gamma wave frequency while decreasing stride speed and swing time variability in PD patients, thereby markedly enhancing neuromotor integration and motor stability (Orcioli-Silva et al., 2025). A 6-month dance intervention significantly enhanced visual system performance, maximal anterior velocity and displacement, and right-side maximal velocity in older adults with mild cognitive impairment. It also significantly shortened posterior reaction time and improved contraction velocity of the rectus femoris and semitendinosus muscles. Moreover, the intervention significantly reduced Falls Efficacy Scale scores and exhibited a trend toward decreased fall incidence, thereby improving postural balance, lower-limb neuromuscular function, and motor confidence, ultimately reducing fall risk in this population (Thiel et al., 2025). A 12-week Pilates training significantly decreased gait and balance scale scores, foot reaction time, and PD Unified Rating Scale motor scores, while increasing step frequency, Berg Balance Scale scores, and functional reach-forward distances, and reducing time required for the stand-up-and-walk timed test in PD patients. These changes collectively enhanced reaction speed, motor function, dynamic and static balance, gait rhythm, and overall mobility (Coban et al., 2025). A 12-week yoga intervention significantly reduced Geriatric Depression Scale and Caregiver Burden Scale scores in AD patients, while enhancing Montreal Cognitive Assessment total scores, verbal ability, attention, delayed recall, abstract thinking, and orientation. Furthermore, the intervention improved depressive symptoms, multiple cognitive domains, overall quality of life, social activity participation, and health status in AD patients (Kaushik et al., 2025). A 3-month adaptive physical activity program, incorporating endurance training, resistance training, and stretching, significantly reduced PD Unified Rating Scale motor scores and increased 6-minute walking distances in PD patients. Additionally, it improved PD Quality of Life Scale scores, thereby enhancing motor function, cardiorespiratory endurance, and overall quality of life (Zanchet et al., 2025).

In summary, various forms and durations of exercise interventions exert multidimensional and sustained benefits targeting core impairments in patients with NDDs, including cognitive deficits, motor dysfunction, and reduced quality of life. However, research on acute exercise interventions remains limited, with their immediate effects, safety, and intensity-dependent relationships warranting further investigation.

## 5 Potential mechanisms of exercise-modulated ferroptosis in neurodegenerative diseases

**FIGURE 2:**
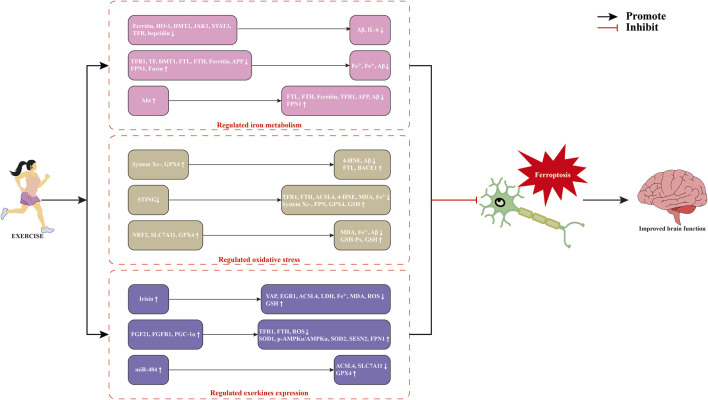
Potential mechanisms of exercise-modulated ferroptosis in neurodegenerative diseases. Abbreviations: 4-HNE, 4-hydroxynonenal; ACSL4, acyl-CoA synthetase long-chain family member 4; Akt, protein kinase B; APP, amyloid precursor protein; Aβ, β-amyloid protein; BACE1, β-site amyloid precursor protein cleaving enzyme 1; DMT1, divalent metal transporter 1; EGR1, early growth response 1; FGF21, fibroblast growth factor 21; FPN, ferroportin; FTH, ferritin heavy chain; FTL, ferritin light chain; GPX4, glutathione peroxidase; GSH, glutathione; HO-1, heme oxygenase-1; IL-6, lnterleukin-6; JAK1, janus kinase 1; LDH, lactate dehydrogenase; MDA, malondialdehyde; miR-484, microRNA-484; NRF2, nuclear factor-erythroid 2 related factor 2; PGC-1α, peroxisome proliferator-activated receptor γ coactivator-1α; ROS, reactive oxygen species; SESN2, sestrin 2; SLC7A11, solute carrier family 7 member 11; SOD1, superoxide dismutase1; STAT3, signal transducer and activator of transcription 3; TF, transferrin; TFR, transferrin receptor protein; YAP, Yes-associated protein.

Exercise is regarded as a cornerstone for disease prevention and treatment, serving as a non-pharmacological intervention alongside conventional therapies for NDDs. Numerous studies have demonstrated that exercise promotes brain iron homeostasis and inhibits ferroptosis by regulating iron transport, antioxidant defenses, and lipid metabolism, thereby maintaining neural function and metabolic stability (Kordi et al., 2024; Thirupathi et al., 2024). The role of exercise in modulating ferroptosis to improve NDDs is definite (Figure 2), but its molecular mechanism still needs to be further elucidated.

### 5.1 Exercise regulates iron metabolism

Iron levels in specific organs such as the brain, skeletal muscles, intestines, and liver gradually increase with age (Chen and Xiao, 2014; Hashimoto et al., 2017). Abnormal iron accumulation induces neuronal ferroptosis and exacerbates oxidative damage, thereby contributing to the development and progression of NDDs (Dusek et al., 2022). Iron homeostasis is primarily maintained through the precise regulation of iron metabolism-related proteins (Ward et al., 2014). Dysregulation of these proteins disrupts systemic iron balance and induces neuronal damage (Ward et al., 2014). Six months of voluntary wheel running significantly downregulated the expression of Ferritin, HO-1, janus kinase 1 (JAK1), signal transducer and activator of transcription 3 (STAT3), DMT1, TFR, and hepcidin in the cerebral cortex of five familial Alzheimer’s disease (5×FAD) mice, reduced Aβ and IL-6 levels, inhibited Aβ plaque deposition and neuroinflammation, thereby improving neurological deficits in AD mice (Belaya et al., 2021). An 8-week treadmill exercise at 70%–85% VO_2_max significantly downregulated the levels of TFR1, TF, DMT1, FTL, FTH, mitochondrial Ferritin, β-secretase, and APP in the cortical motor regions of aged amyloid precursor protein-C105 (APP-C105) mice, upregulated the expression of FPN1, Furin, and α-secretase, significantly reduced Fe^2+^, Fe^3+^, and total iron content, inhibited brain iron accumulation and Aβ plaque growth, thereby improving learning ability, memory, and cognitive function in aged APP-C105 mice (Choi et al., 2021). Fifty-five days of swimming training markedly activated the Akt signaling pathway in the skeletal muscle of SOD1^G93A^ transgenic mice, significantly reduced the expression of FTL, FTH, Ferritin, TFR1, and APP, upregulated FPN1 expression, inhibited Aβ and iron accumulation in skeletal muscle, and improved neural function in the mice. *In vitro* experiments demonstrated that Akt inhibition markedly upregulated the expression of FTL, FTH, TFR1, and Poly (rC)-binding protein 1 (PCBP1), downregulated FPN1 expression, increased APP expression, enhanced iron storage and uptake, and decreased iron efflux, thereby aggravating neurological injury in SH-SY5Y and C2C12 cells (Halon-Golabek et al., 2024). Therefore, exercise interventions may regulate iron metabolism-related protein expression, influence systemic iron homeostasis, and consequently delay the pathological progression of NDDs.

### 5.2 Exercise regulates oxidative stress

As a principal driver of ferroptosis, oxidative stress is defined as a physiological or pathological condition wherein the production of ROS and reactive nitrogen species (RNS) under endogenous or exogenous stimuli surpasses the neutralising capacity of the antioxidant defence system, thereby causing oxidative damage (Islam, 2017). Excessive iron accumulation in the brain intensifies oxidative stress, amplifies lipid peroxidation, and promotes ferroptosis, thereby accelerating neuronal degeneration (Zhang et al., 2024). Eight weeks of aerobic exercise significantly activated the System Xc^−^/GPx4 signaling pathway in the prefrontal cortex of APPswe/PS1dE9 transgenic AD mice, up-regulated FTL and BACE1 expression, significantly downregulated 4-HNE levels, inhibited oxidative stress, lipid peroxidation, and ferroptosis, reduced Aβ positivity, alleviated neuronal morphological abnormalities, and improved learning and memory functions in AD mice (Li et al., 2024a). Four weeks of moderate-intensity treadmill exercise significantly inhibited the activation of the STING signaling pathway in the damaged cortex of traumatic brain injury mice, downregulated the expression of TFR1, FTH, and ACSL4, upregulated FPN, System Xc^−^, GPX4, and GSH expression, and reduced levels of 4-HNE, MDA, and Fe^2+^. This intervention alleviated abnormalities in iron homeostasis, lipid peroxidation, and antioxidant system damage, inhibited ferroptosis, and improved neurological damage and cognitive dysfunction. Additionally, overexpression of STING reversed the inhibitory effect of exercise on ferroptosis, thereby exacerbating neurological damage (Chen et al., 2023). Fourteen days of treadmill training significantly enhanced the expression of NRF2, GPX4, and SLC7A11 in the cerebral cortex of rats with cerebral ischemia-reperfusion injury. It activated the SLC7A11/GPX4 signaling pathway, upregulated GSH-Px and GSH expression, decreased MDA and ferroptosis levels, and inhibited lipid peroxidation and ferroptosis in the cerebral cortex, thereby improving motor function, balance, and neurological deficits in the rats. Erastin, a ferroptosis activator, significantly attenuated the neuroprotective effects of treadmill training in rats with cerebral ischemia-reperfusion injury (Liu et al., 2022b). Therefore, aerobic exercise can modulate oxidative stress in brain tissue, inhibit lipid peroxidation and ferroptosis, and mitigate neurological deficits.

### 5.3 Exercise regulates exerkines expression

Exerkines are bioactive molecules secreted by exercise-stimulated tissues such as skeletal muscle, adipose tissue, liver, brain, and other organs, including myokines, cardiokines, hepatokines, adipokines, neurokines, lactate, and miRNAs. These exerkines act through autocrine, paracrine, or endocrine mechanisms to mediate inter-organ communication, playing essential roles in regulating nervous system function, attenuating neuroinflammation, and promoting neural repair (Chow et al., 2022; Pedersen and Fischer, 2007). Studies have confirmed that Irisin is a protein-like myokine secreted during exercise, derived from fibronectin type III domain-containing protein 5 (FNDC5) (Ma et al., 2025; Akbulut et al., 2025). Irisin-loaded bone marrow mesenchymal stem cell-derived exosomes significantly downregulated the expression of Yes-associated protein (YAP), early growth response 1 (EGR1), and ACSL4, inhibited activation of the YAP/EGR1/ACSL4 signaling pathway, significantly increased GSH levels, decreased levels of Fe^2+^, lactate dehydrogenase (LDH), MDA, and lipid ROS, inhibited neuronal ferroptosis and lipid peroxidation, thereby ameliorating oxygen-glucose deprivation and reoxygenation (OGD/R)-induced neuronal injury. *In vivo* experiments further demonstrated that Irisin-loaded bone marrow mesenchymal stem cell-derived exosomes significantly inhibited activation of the YAP/EGR1/ACSL4 signaling pathway in the brains of middle cerebral artery occlusion (MCAO) mice, suppressed ferroptosis and lipid peroxidation, thereby mitigating ischemic brain injury (Fang et al., 2025). Six weeks of aerobic exercise significantly upregulated the expression of fibroblast growth factor 21 (FGF21), FGFR1, and peroxisome proliferator-activated receptor γ coactivator-1alpha (PGC-1α) in the paraventricular nucleus of mice with myocardial ischemia/reperfusion injury, increased the phosphorylated AMP-activated protein kinase alpha (p-AMPKα)/AMPKα ratio, upregulated the expression of SOD1, SOD2, and sestrin 2 (SESN2), reduced ROS levels, downregulated the expression of TFR1 and FTH, upregulated the expression of FPN1, suppressed neuronal oxidative stress and ferroptosis, thereby ameliorating neuronal injury in mice with myocardial ischemia/reperfusion injury. *In vitro* experiments further confirmed that FGF21 protected HT22 cells from OGD/R-induced oxidative stress and ferroptosis, thereby attenuating neuronal injury (Zhao et al., 2025c). OGD-induced ACSL4 expression was significantly elevated, while the expression of GPX4 and SLC7A11 was significantly reduced in PC12 cells. Furthermore, ROS and LDH levels were increased, promoting ferroptosis in PC12 cells. *In vivo* experiments confirmed that 4 weeks of aerobic training significantly increased the expression of exosome miR-484 in the skeletal muscle of ischemic stroke rats. Exosome miR-484 in skeletal muscle was found to target and downregulate ACSL4 expression, upregulate GPX4 and SLC7A11 expression in brain tissue, inhibit neuronal ferroptosis and lipid peroxidation, and promote nerve repair (Huang et al., 2024). Therefore, exercise regulates the expression of various exerkines, inhibits neuronal ferroptosis, and exerts neuroprotective effects.

## 6 Conclusion and perspective

Iron, an essential trace element and pivotal signaling molecule in the nervous system, can drive ferroptosis, which contributes to the pathogenesis of NDDs such as AD, PD, MS, and ALS. Targeting ferroptosis has emerged as a promising strategy for the prevention and treatment of NDDs. Exercise, as a vital non-pharmacological intervention, holds great promise in preventing, treating, and rehabilitating NDDs, as it can directly or indirectly suppress ferroptosis and mitigate NDD progression by regulating iron metabolism, oxidative stress, and exerkines expression.

Currently, research on the role of ferroptosis in NDDs and the potential mechanisms of exercise interventions remains limited. Several critical questions remain unresolved: (1) most existing studies are confined to animal and cellular models, with a notable lack of large-scale population-based investigations and clinical validation, thereby impeding translational application; (2) although iron chelators and antioxidants have been used to modulate ferroptosis, their therapeutic efficacy remains inadequate, limiting their clinical utility; and (3) whether different exercise modalities and intensities exert differential effects on ferroptosis, and how these variations influence NDDs progression, has yet to be elucidated. Addressing these questions in future research will help establish a mechanistic basis for the neuroprotective effects of exercise and its implications for brain health.
