# Supplementary material for: Ferroptosis in neurodegenerative diseases: potential mechanisms of exercise intervention
Source: Front Cell Dev Biol. 2025 Jun 30;13:1622544. doi: 10.3389/fcell.2025.1622544 (PMC12256474; doi:10.3389/fcell.2025.1622544)
Supplement: Supplementary file 1 [file DataSheet1.docx]

Supplementary Material

# Supplementary Tables and Figures

## Table 1

**TABLE 1** Role of ferroptosis in neurodegenerative diseases

| **NDDs** | **Compounds/Drugs/Proteins/RNA** | **ferroptosis** | **Marker proteins** | **Main functions** | **Refs** |
| --- | --- | --- | --- | --- | --- |
| AD | Ghrelin ↑ | ↓ | BMP6, SMAD1, SLC7A11, GPX4, FTL1, FTH1, Arg-1, IL-10, TGF-β ↑ | Promoted the polarization of microglia towards M2, and improved learning and memory impairments in AD mice | (Guo Y et al., 2025) |
|  | Neuritin ↑ | ↓ | Map2, NeuN, PSD95, GSH, NADPH ↑  ROS, MDA, 4-HNE ↓ | Enhanced neuronal signal transmission and synaptic plasticity, inhibited neuronal oxidative stress, and improved cognitive impairment and learning and memory abilities in AD mice | (Song et al., 2025) |
|  | GAA ↑ | ↓ | GPX4, SLC7A11, NRF2, FTH1, SOD, GSH ↑  Fe^2+^, TFR1, ACSL4, MDA, GSSG ↓ | Improved the learning and memory abilities of AD mice | (Lu et al., 2025) |
|  | Artemisinin ↑ | ↓ | NRF2, SLC7A11, GPX4, GSH ↑  KEAP1, MDA, ROS ↓ | Improved the learning and memory abilities of AD mice | (Deng et al., 2025) |
| PD | Neural stem cell-derived exosomes CDC42 ↑ | ↓ | GSH, GPX4, VEGF, IL-8 ↑  ACSL4, ROS, MDA, 4-HNE, Fe^2+^, α-Syn ↓ | Reduced cerebral vascular damage and cognitive and memory dysfunction in PD mice | (Li Y et al., 2025) |
|  | TIGAR ↑ | ↓ | NADPH, GPX4, GSH, ↑  GSSG, Fe^2+^, MDA, ROS ↓ | Inhibited ferroptosis in dopaminergic neurons and improved neurological function | (Sheng et al., 2025) |
|  | FTO ↓ | ↓ | YTHDF2, SLC7A11 ↑  BAP1, α-Syn, MDA, Fe^2+^, 4-HNE ↓ | Improved the health of dopaminergic neurons in PD mice | (Li Z et al., 2025) |
|  | IRP2 ↓ | ↓ | SLC7A11, GPX4 ↑  p53, TFR1, FTH, ALOX12 ↓ | Reduced PC-12 cell damage | (Yao et al., 2024) |
| MS | MAT ↑ | ↓ | GSH, SOD, GPX4, SLC7A11 ↑  MDA, LPCAT3, PTGS2, IL-6, TNF-α, IL-1β ↓ | Reduced neuroinflammation and central nervous system damage in EAE mice | (Feng et al., 2024) |
|  | Dabrafenib ↑ | ↓ | Axl, System Xc⁻, GPX4, Ferritin ↑  CD71, ACSL4, POR, Iba-1, ROS ↓ | Improved gait abnormalities and limb weakness in EAE mice | (Liu et al., 2024) |
|  | Bone marrow mesenchymal stem cell-derived exosomes miR-367-3p ↑ | ↓ | SLC7A11, GPX4, GSH, SOD↑  EZH2, Fe^2+^, MDA, ↓ | Reduced inflammation and injury to the spinal cord | (Fan et al., 2023) |
|  | DFP ↑ | ↓ | GSH, NRF2, GPX4 ↑  Fe^2+^, TFR1, Iba-1 ↓ | Reduced demyelination and optic nerve damage in mice | (Rayatpour et al., 2022) |
| ALS | 25-OHC ↑ | ↑ | PTGS2, ROS, CYB5R1, POR ↑  SREBP, GXP4, SCD1, GSH ↓ | aggravated damage to glial cells | (Urano et al., 2025) |
|  | NRF2 ↓ | ↑ | MDA, GSH, ROS ↑  SLC7A11, GPX4 ↓ | aggravated motor neuron damage | (Yang et al., 2023) |
|  | SPY1 ↓ | ↑ | ALOX15, GDF15, TFR1, Fe^2+^ ↑  GCH1, GPX4, GSH ↓ | Exacerbated muscle atrophy and motor dysfunction in mice | (Wang et al., 2023) |
|  | MPO ↑ | ↑ | HOCl , Caspase-3, MDA ↑  GPX4, NQO1 ↓ | Reduces motor performance in mice | (Peng et al., 2022) |

Abbreviations: BMP6: bone morphogenetic protein 6; SLC7A11: solute carrier family 7 member 11; GPX4: glutathione peroxidase; FTL1: ferritin light chain; FTH1: ferritin heavy chain 1; Arg-1: arginase 1; IL-10: lnterleukin-10; TGF-β: transforming growth factor-β; Map2: microtubule-associated protein 2; NeuN: neuronal nuclei; PSD95: postsynaptic density protein 95; GSH: glutathione; NADPH: nicotinamideadenine dinucleotide phosphate; ROS: reactive oxygen species; MDA: malondialdehyde; GAA: ganoderic acid A; 4-HNE: 4-hydroxynonenal; NRF2: nuclear factor-erythroid 2 related factor 2; SOD: superoxide dismutase; TFR1: transferrin receptor protein 1; ACSL4: acyl-CoA synthetase long-chain family member 4; GSSG: oxidized glutathione; KEAP1: kelch-like ECH-associated protein 1; VEGF: vascular endothelial growth factor; TIGAR: Tp53-induced glycolysis and apoptosis regulator; α-Syn: α-Synucleinα; FTO: fat mass and obesity- associated protein; YTHDF2: YTH domain family protein 2; BAP1: BRCA1-associated protein 1; IRP2: iron regulatory protein 2; ALOX12: arachidonate 12-Lipoxygenase; MAT: matrine; LPCAT3: lysophosphatidylcholine acyltransferase 3; PTGS2: prostaglandin- endoperoxide synthase 2; TNF-α: tumor necrosis factor alpha; EAE: experimental autoimmune encephalomyelitis; Axl: Axl receptor tyrosine kinase; POR: cytochrome P450 oxidoreductase; Iba-1: ionized calcium-binding adapter molecule 1; EZH2: enhancer of zeste homolog 2; DFP: deferiprone; 25-OHC: 25-hydroxycholesterol; CYB5R1: cytochrome b5 reductase 1; SREBP: sterol regulatory element-binding protein; SCD1: stearoyl-CoA desaturase 1; SPY1: speedy/RINGO cell cycle regulator family member A; GDF15: growth differentiation factor 15; GCH1: GTP cyclohydrolase 1; MPO: myeloperoxidase; HOCl: hypochlorous acid; Caspase-3: cystein-asparate protease 3; NQO1: quinone oxidoreductase 1; NDDs: neurodegenerative diseases; Refs: References; AD: alzheimer's disease; PD: parkinson's disease; MS: multiple sclerosis; ALS: amyotrophic lateral sclerosis.

## Figure 1


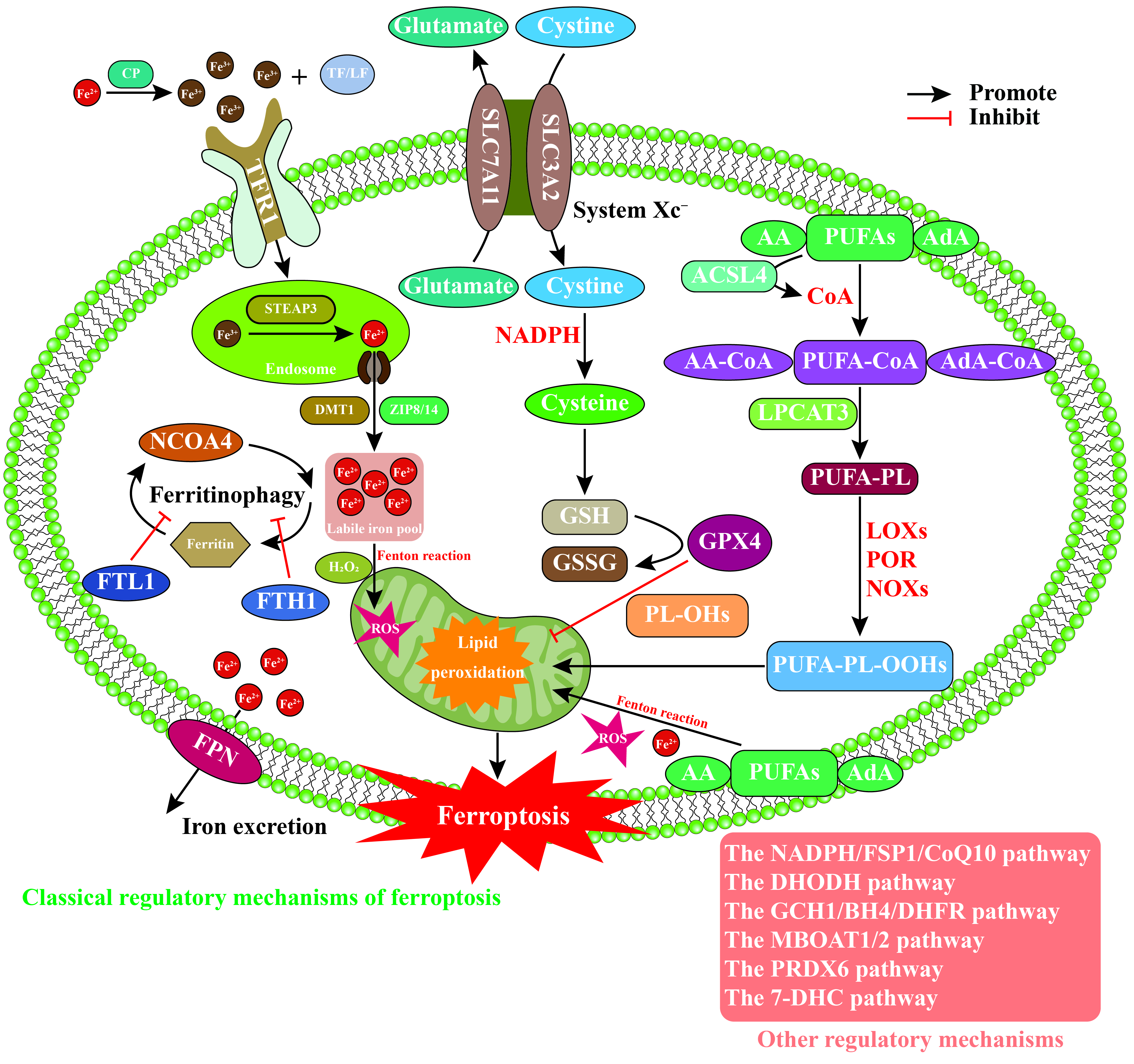


**FIGURE 1** Regulatory mechanisms of ferroptosis. Abbreviations: 7-DHC: 7-dehydrocholesterol; AA: arachidonic acid; ACSL4: acyl-CoA synthetase long-chain family member 4; AdA: adrenic acid; BH4: tetrahydrobiopterin; CoA: coenzyme A; DHFR: dihydrofolate reductase; DHODH: dihydroorotate dehydrogenase; DMT1: divalent metal transporter 1; FPN: ferroportin; FSP1: ferroptosis suppressor protein 1; FTH1: ferritin heavy chain 1; FTL1: ferritin light chain 1; GCH1: GTP cyclohydrolase 1; GPX4: glutathione peroxidase; GSH: glutathione; LF: lactoferrin; LOXs: lipoxygenases; LPCAT3: lysophosphatidylcholine acyltransferase 3; MBOAT1/2: membrane-bound O-acyltransferase 1/2; NADPH: nicotinamideadenine dinucleotide phosphate; NCOA4: nuclear receptor coactivator 4; NOXs: NADPH oxidases; PL-OHs: phospholipid alcohols; POR: cytochrome P450 oxidoreductase; PRDX6: peroxiredoxin 6; PUFA-PL-OOHs: polyunsaturated fatty acid-containing phospholipid hydroperoxides; PUFA-PLs: polyunsaturated fatty acid–containing phospholipids; PUFAs: polyunsaturated fatty acids; ROS: reactive oxygen species; SLC3A2: solute carrier family 3 member 2; SLC7A11: solute carrier family 7 member 11; STEAP3: six-transmembrane epithelial antigen of prostate 3; TF: transferrin; TFR1: transferrin receptor protein 1.

## Figure 2


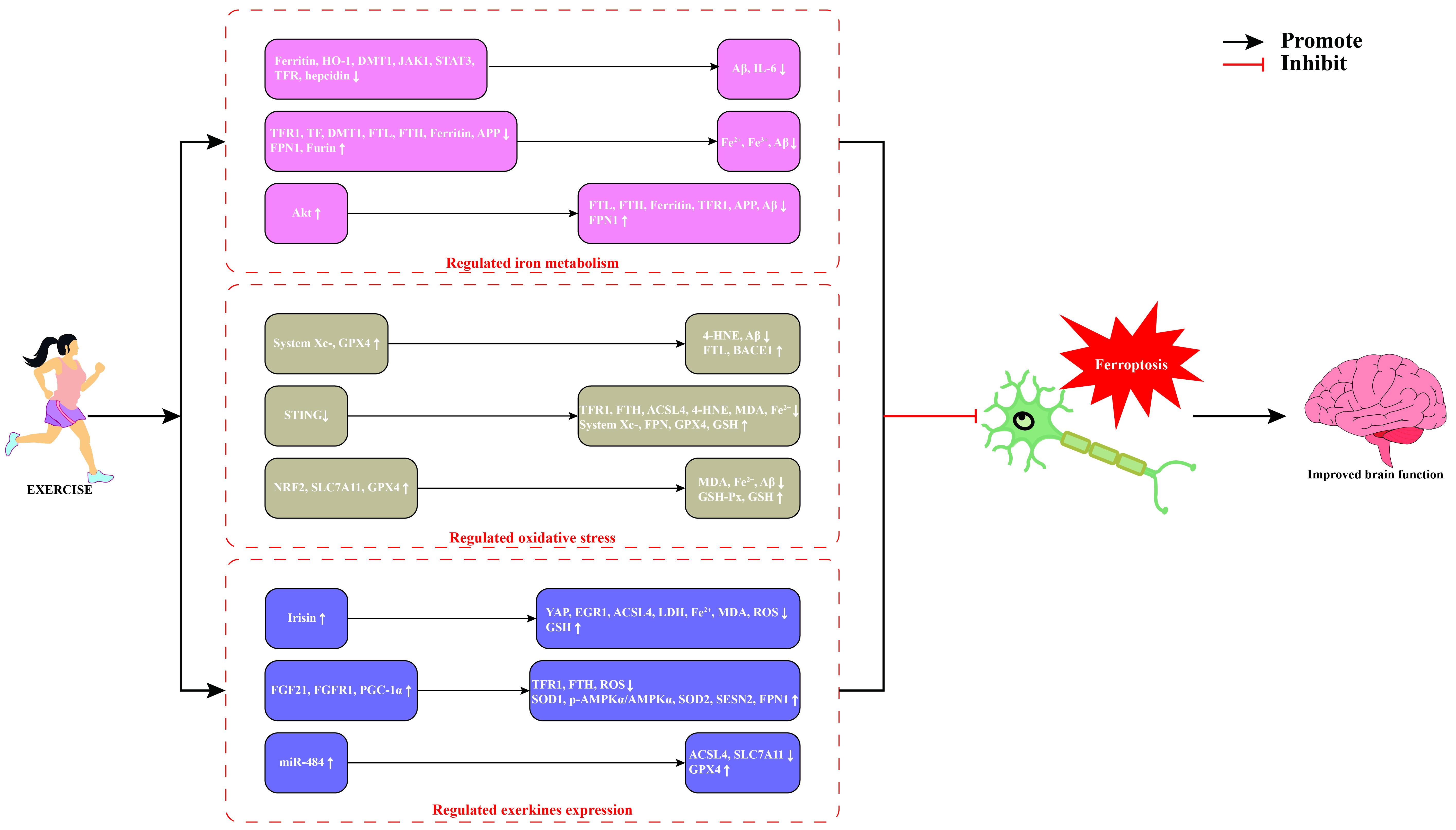


**FIGURE 2** Potential mechanisms of exercise-modulated ferroptosis in neurodegenerative diseases. Abbreviations: 4-HNE: 4-hydroxynonenal; ACSL4: acyl-CoA synthetase long-chain family member 4; Akt: protein kinase B; APP: amyloid precursor protein; Aβ: β-amyloid protein; BACE1: β-site amyloid precursor protein cleaving enzyme 1; DMT1: divalent metal transporter 1; EGR1: early growth response 1; FGF21: fibroblast growth factor 21; FPN: ferroportin; FTH: ferritin heavy chain; FTL: ferritin light chain; GPX4: glutathione peroxidase; GSH: glutathione; HO-1: heme oxygenase-1; IL-6: lnterleukin-6; JAK1: janus kinase 1; LDH: lactate dehydrogenase; MDA: malondialdehyde; miR-484: microRNA-484; NRF2: nuclear factor-erythroid 2 related factor 2; PGC-1α: peroxisome proliferator-activated receptor γ coactivator-1α; ROS: reactive oxygen species; SESN2: sestrin 2; SLC7A11: solute carrier family 7 member 11; SOD1: superoxide dismutase1; STAT3: signal transducer and activator of transcription 3; TF: transferrin; TFR: transferrin receptor protein; YAP: Yes-associated protein.
